# Functional Roles of JNK and p38 MAPK Signaling in Nasopharyngeal Carcinoma

**DOI:** 10.3390/ijms23031108

**Published:** 2022-01-20

**Authors:** Lesley Jia Wei Pua, Chun-Wai Mai, Felicia Fei-Lei Chung, Alan Soo-Beng Khoo, Chee-Onn Leong, Wei-Meng Lim, Ling-Wei Hii

**Affiliations:** 1 School of Postgraduate Studies, International Medical University, Bukit Jalil, Kuala Lumpur 57000, Malaysia; 00000011996@student.imu.edu.my (L.J.W.P.); coleong@agtcgenomics.com (C.-O.L.); 2 Center for Cancer and Stem Cell Research, Development and Innovation (IRDI), Institute for Research, International Medical University, Bukit Jalil, Kuala Lumpur 57000, Malaysia; mai.chunwai@outlook.com (C.-W.M.); alk2003@alumni.weill.cornell.edu (A.S.-B.K.); 3 Department of Medical Sciences, School of Medical and Life Sciences, Sunway University, Bandar Sunway 47500, Malaysia; feliciacfl@sunway.edu.my; 4 AGTC Genomics, Bukit Jalil, Kuala Lumpur 57000, Malaysia; 5 School of Pharmacy, International Medical University, Bukit Jalil, Kuala Lumpur 57000, Malaysia

**Keywords:** p38 mitogen-activated protein kinase, c-Jun N-terminal kinase, nasopharyngeal carcinoma, Epstein–Barr virus, cancer cell survival

## Abstract

c-Jun N-terminal kinase (JNK) and p38 mitogen-activated protein kinase (MAPK) family members integrate signals that affect proliferation, differentiation, survival, and migration in a cell context- and cell type-specific way. JNK and p38 MAPK activities are found upregulated in nasopharyngeal carcinoma (NPC). Studies have shown that activation of JNK and p38 MAPK signaling can promote NPC oncogenesis by mechanisms within the cancer cells and interactions with the tumor microenvironment. They regulate multiple transcription activities and contribute to tumor-promoting processes, ranging from cell proliferation to apoptosis, inflammation, metastasis, and angiogenesis. Current literature suggests that JNK and p38 MAPK activation may exert pro-tumorigenic functions in NPC, though the underlying mechanisms are not well documented and have yet to be fully explored. Here, we aim to provide a narrative review of JNK and p38 MAPK pathways in human cancers with a primary focus on NPC. We also discuss the potential therapeutic agents that could be used to target JNK and p38 MAPK signaling in NPC, along with perspectives for future works. We aim to inspire future studies further delineating JNK and p38 MAPK signaling in NPC oncogenesis which might offer important insights for better strategies in diagnosis, prognosis, and treatment decision-making in NPC patients.

## 1. Introduction

Nasopharyngeal carcinoma (NPC) is one of the most aggressive types of head and neck carcinoma that mainly grows at the epithelial lining of the nasopharynx, with frequent metastasis to regional lymph nodes and occasionally to distal organs [1,2,3]. Compared with other cancer types, NPC is characterized by its unique epidemiological feature where significantly higher incidence rates were observed in endemic regions, such as Southeast Asia and southern China (21 cases per 100,000 population), compared to the Western countries (1 case per 100,000 population) [4]. Several risk factors, including genetic predisposition, viral infection (e.g., Epstein–Barr virus and human papillomavirus), and diet, have been shown to associate with NPC pathogenesis [5,6,7,8]. Despite its high sensitivity to current treatments, such as ionizing radiotherapy, if treated at early stages treatment is often not curative, as approximately 10% to 20% of NPC patients will have local recurrence, while about 7 to 20% will have distant metastasis within 2 years after primary therapy [9,10,11].

**Figure 1:**
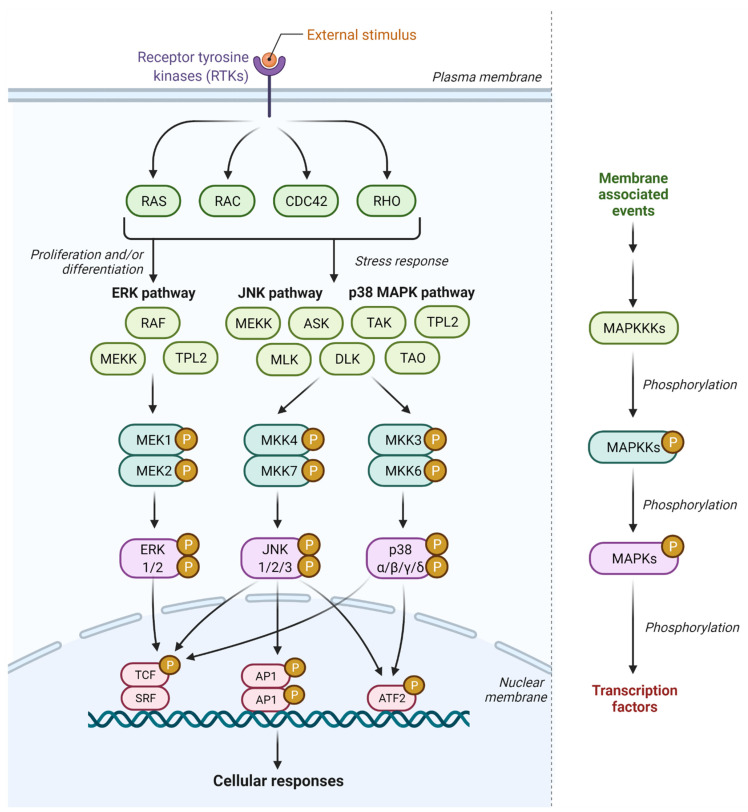
JNK, p38 MAPK, and ERK pathways in MAPK signaling. Upon external stimulation, receptor tyrosine kinases (RTKs) activate the three-tiered kinase module comprising MAPKK,. MAPKK, and MAPK through sequential protein phosphorylation. The activated MAPKs translocate to the nucleus and trigger cellular responses.

Mitogen-activated protein kinase (MAPK) pathway is an intracellular signal transduction pathway that regulates a plethora of cellular processes, including cell growth, cell proliferation, cell differentiation, stress response, migration, and apoptosis, in response to various extracellular stimuli [12,13,14]. It consists of three pathways which involve extracellular-signal-regulated kinase 1 and 2 (ERK1/2), c-Jun N-terminal kinase 1, 2, and 3 (JNK1/2/3), and p38 MAPK signaling pathways (Figure 1) [15]. ERK1/2 is activated in response to growth factors, hormones, and proinflammatory stimuli, while JNK1/2/3 and p38 MAPKs are activated by cellular and environmental stresses, in addition to proinflammatory stimuli [15].

MAPKs are activated upon binding of the ligands to the transmembrane glycoproteins of the receptor tyrosine kinase (RTK) family proteins, such as epidermal growth factor receptor (EGFR), fibroblast growth factor receptor (FGFR), platelet-derived growth factor receptor (PDGFR), and vascular endothelial growth factor receptor (VEGFR) [16,17]. Alternatively, some RTKs may be activated in a ligand-independent manner by external stimuli or through activating mutations (as in cancers). The activation signal is then transmitted through rat sarcoma (RAS) signaling molecules via rapid conversion of GTP to GDP with the aid of nucleotide exchange factor Son of Sevenless homolog 1 (SOS1) [18]. The active form of RAS will then bind to RAF proto-oncogene serine/threonine-protein kinase (rapidly accelerated fibrosarcoma, RAF) and activates the mitogen-activated protein kinase kinase kinases (MAPKKK), the mitogen-activated protein kinase kinases (MAPKK), and the MAPKs in sequential order (Figure 1) [13,19]. This in turn leads to the phosphorylation of the MAPK substrates, such as phospholipases, transcription factors, and cytoskeletal proteins, at their specific interaction motifs, leading to the upregulation or downregulation of the target genes [13,20].

The MAPK family proteins have attracted researchers’ attention due to their significant role in regulating cancer cell survival. They have been frequently reported to be involved in oncogenesis, tumor progression, and drug resistance [21]. While MAPKs play crucial roles in apoptosis and cancer cell survival, their functional roles and mechanisms in the oncogenesis of NPC are less understood. In the context of NPC, only the ERK pathway has been extensively studied and reviewed, while findings and literature on JNK and p38 MAPK pathways in NPC remain fragmented [22]. Therefore, this narrative review begins with an overview of JNK and p38 MAPK pathways in human cancers, followed by a focused discussion on the functions of JNK and p38 MAPK signaling pathways in NPC. This review also summarizes the potential therapeutics that could be used to target JNK and p38 MAPK signaling in NPC, along with perspectives for future works to address the research gaps.

## 2. JNK Signaling Pathway

JNK is one of the MAPK family proteins that is predominantly activated by stress stimuli. Initially, the JNK cascade was found in mice liver and called stress-activated protein kinase (SAPKs) but was later named JNK due to its ability to phosphorylate and activate the c-Jun transcription factor [23]. There are three JNK isoforms, encoded by three genetic loci known as JNK1 (MAPK8), JNK2 (MAPK9), and JNK3 (MAPK10 [24]. JNK1 and JNK2 are found in most tissues, while JNK3 expression is limited to the tissues of the brain, heart, and testes [25].

**Figure 2:**
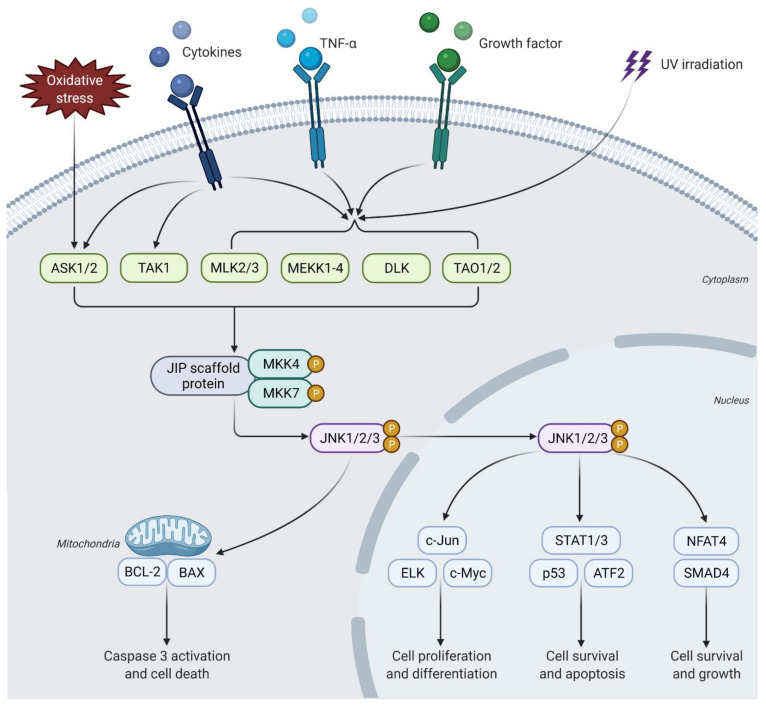
The upstream activators and downstream targets of the JNK pathway. Several types of stimuli, such as inflammatory cytokines, tumor necrosis factor alpha (TNF-α), and growth factors, can induce activation of members of the MAPKKK family. Other factors such as oxidative stress and UV irradiation can also lead to MAPKKK activation. The scaffold protein JNK-interacting protein-1 (J1P) binds the MAPKKK and MAPKK family members with JNKs and facilitates the JNK activation. The activated JNKs may dissociate from this complex and induce mitochondria-dependent apoptosis through B cell lymphoma (BCL-2) and BCL-2 associated x-protein (BAX). On the other hand, activated JNKs may also promote transcription of genes involved in cell proliferation, differentiation, growth, and apoptosis via phosphorylation of the downstream targets.

JNK is activated by a series of phosphorylation events. Upon stimuli activation, phosphorylation of MAPKKK (e.g., MEKK1-4, ASK1/2, TAK1, MLK2, DLK, and TAO1/2) leads to phosphorylation of MAPKK (e.g., MKK4 and MKK7), which in turn leads to dual phosphorylation of JNKs at the threonine (Thr183) and tyrosine (Tyr185) sites on its Thr-Pro-Tyr (TPY) motif [26,27]. Activated JNKs will then phosphorylate Jun proteins (JunB, JunD, and c-Jun), which leads to its dimerization with Fos proteins (c-Fos, FosB, Fra-1/2) to form the transcription factor activator protein-1 (AP-1) to in turn activate the transcriptional program of the target genes [26,27]. Activated JNKs can also regulate the transcription of c-Myc, p53, ETS Like-1 protein (ELK1), activating transcription factor 2 (ATF2), nuclear factor of activated T cell (NFAT), signal transducer and activator of transcription 1/3 (STAT1/3), paired box (PAX) genes, and the BCL2 family proteins (e.g., BCL2, BCL-xL, BAD, BIM, and BAX) [26,27]. As shown in Figure 2, these signals eventually lead to the regulation of multiple cellular processes, including cell proliferation and apoptosis, immunological effects, insulin signaling, and neuronal activity [26,27,28].

## 3. p38 MAPK Signaling Pathway

p38 MAPK is another type of SAPK under the MAPK family which is primarily activated by environmental stress (e.g., heat, osmotic, and oxidative stress) and genotoxic stress (e.g., ionizing radiation, ultraviolet (UV) light, and cytotoxic DNA damaging agents) [29,30]. The p38 MAPKs consist of four isoforms encoded by separate genes with distinct substrate specificities and tissue distributions: p38α (MAPK14), p38β (MAPK11), p38γ (MAPK12), and p38δ (MAPK13) [13].

**Figure 3:**
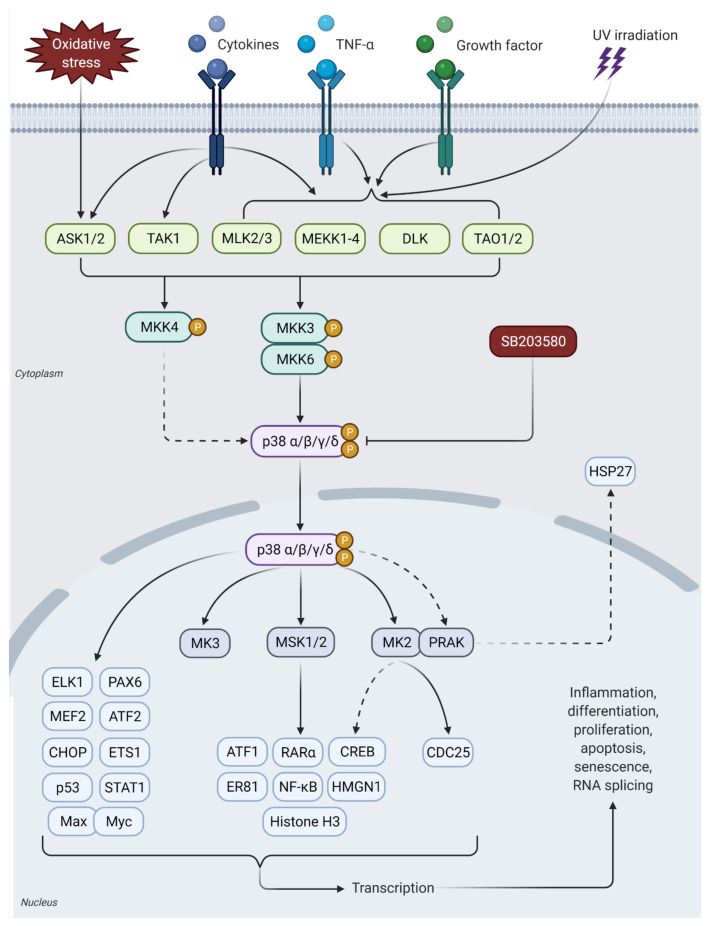
p38 MAPKs pathway and its upstream and downstream activation. The four p38 MAPK family members (p38α, p38β, p38γ, and p38δ) are activated by external stress, inflammatory cytokines, or UV radiation, which is similar to the JNK pathway. Once activated, this pathway initiates production of the pro-apoptotic transcription factors for inflammation, differentiation, proliferation, apoptosis, senescence, and RNA splicing.

Different isoforms of p38 are differentially expressed in different tissues. For instance, p38α and p38β are ubiquitously expressed in most tissues and are particularly highly expressed in heart and brain; p38γ is expressed primarily in skeletal muscle, while p38δ expression is mainly in the lungs, pancreas, small intestine, kidneys, and testis [31]. The main upstream MAP2Ks involved in p38 MAPK activation are MKK3 and MKK6 and, to a lesser extent, MKK4 [31,32]. As with JNK activation, activation of p38 MAPK requires dual phosphorylation by MAP3Ks at the Thr-Gly-Tyr (TGY) motif [13,33]. Once the p38 MAPKs are activated, they translocate from the cytosol to the nucleus and regulate cellular functions by activating downstream transcriptional targets such as PAX6, ETS1, PRAK, MK3, RARα, AP-1, ATF1, and CHOP [13,34]. Activation of p38 MAPK by MSK1/2 can activate other transcription factors, including STAT1, NF-κB, MEF-2, ELK1, and CREB [13]. In addition, different isoforms of p38 MAPK can specifically activate different kinds of downstream molecules depending on their substrate specificity. For instance, p38α, p38β, and p38γ activate MAPK-activated protein (MAPKAP-2 or MK2) and heat shock protein 27 (HSP27), while p38δ activates eukaryotic elongation factor 2 kinase (eEF2K) [35,36,37,38]. These proteins will ultimately lead to regulation of gene expression, cell motility, transcription, and chromatin remodeling (Figure 3) [19].

## 4. JNK and p38 MAPK Signaling in Human Cancers

In normal tissue, the activation of both JNK and p38 MAPK pathways is triggered mainly by metabolic stress, DNA damage, cytokines, and growth factors, which in turn regulate cell viability [14]. Activation of p38 MAPK signaling has been shown to induce the expression of pro-inflammatory mediators, including cyclooxygenase-2 (COX-2) and tumor necrosis factor-α (TNF-α), while activation of JNK leads to induction of apoptosis in response to stress stimuli or inflammatory or oncogenic signals [31,39].

In cancerous cells, both JNK and p38 MAPK pathways usually exhibit dysregulation of protein expression [40]. Several studies have demonstrated that upregulation of JNK and p38 MAPK signaling enhances tumor growth and cancer cell invasion [41,42,43]. However, a number of studies also indicated that p38 MAPK signaling is downregulated in tumor cells, resulting in the development of anoikis resistance, and promotes survival of circulating cancer cells [44]. Thus, the role of JNK and p38 MAPK signaling in cancers remains controversial. It is suggested that JNK and p38 MAPK signaling could exert an oncosuppressive or oncogenic function in a cell context-dependent manner (Table 1 and Table 2) [26,45].

## 5. Pro-Tumorigenic Functions of JNK and p38 MAPK Signaling in NPC

The activation of JNK and p38 MAPK signaling has been shown to exert pro-tumorigenic functions in NPC by promoting NPC tumor growth, cell invasion, metastasis, and angiogenesis. JNK and p38 MAPK activities are also found to inhibit pro-apoptotic signaling in NPC cells and can be induced by LMP1 in EBV-associated NPC. Moreover, it is suggested that activation of p38 MAPK signaling may contribute to inflammatory tumor microenvironment in NPC.

### 5.1. Activation of JNK Signaling Promotes NPC Cell Survival

Prolonged activation of JNKs has been shown to promote NPC tumorigenesis by activating c-Jun. For instance, increased expression of c-Jun, JNK, phosphorylated c-Jun, and phosphorylated JNK protein was associated with tumor (T), nodes (N), and metastases (M) staging and was expressed at significantly higher levels in patients with stage III–IV than those with I–II stage NPC [59]. A study has also found that c-Jun silencing showed a significant drop in cell migration and invasion both in vitro and in vivo [79]. As an important part of AP-1 transcription factor, c-Jun plays a major interactive role in tumor formation, invasion, metastasis, and production of various cytokines and growth factors in NPC [59]. It is reported that increased expression of serine/threonine phosphatase calcineurin (CaN) could increase the half-life of c-Jun proteins, resulting in high expression of c-Jun, followed by enhancement of tumorigenesis [80]. In addition, prolonged JNK activation in NPC also has been found to increase the phosphorylation and subsequent deactivation of p53, resulting in the activation of DNA methyltransferase and increased resistance to apoptosis [22].

### 5.2. JNK and p38 MAPK Activities Inhibit Pro-Apoptotic Signaling in NPC Cells

As mentioned in Table 1 and Table 2, increased expression of p38 MAPKs and JNKs has also been reported in NPC [59,78]. JNK and p38 MAPK activities have been evaluated in treatment-resistant NPC, of which the protein expressions of both JNK and p38 MAPK were found to be higher in cetuximab-resistant NPC [81]. Stroma cell-derived factor 1 (SDF-1) is known to stimulate the activation of the p38 MAPK pathway in NPC cells with downregulation of microRNA-9 (miR-9), which reportedly exhibits tumor suppressor properties, through CXCR4 overexpression, leading to NPC cell growth, migration, and invasion [82]. 

Dysregulation of apoptotic signals is one of the hallmark factors for the development of various types of human cancer, including NPC. The well-known case is the aberrant activation of BCL2 [22]. Overexpressed BCL2 protein in NPC has been reported in a higher percentage than other head and neck cancers [83]. The upregulation of BCL2 mRNA has also been found in several studies in NPC biopsies [84,85]. JNK signaling has been shown to regulate the activity of BCL2 and anti-apoptosis activity in NPC. A study demonstrated that JNK inhibition by programmed cell death 4 (PDCD4), a tumor suppressor gene, could inhibit BCL2 activity, resulting in the blockade of cell proliferation and cell cycle progression as well as inducing the mitochondrial apoptosis pathway [86]. JNK activation stimulated by TNF-α upregulated the expression of inhibitor of apoptosis protein 2 (c-IAPs 2), which then triggered rapid proliferation of NPC [87]. In addition, it has been reported that treatment with 14-thienyl methylene matrine (YYJ8) inhibited the proliferation of NPC cell lines and induced apoptosis by suppressing p38 phosphorylation followed by BCL-2 activation [88].

### 5.3. JNK and p38 MAPK Signaling Mediates LMP1 in EBV-Associated NPC

Compared to other head and neck cancers, NPC possesses a unique relationship with the Epstein-Barr virus (EBV). EBV-associated NPC is the most prevalent and most invasive form of head and neck carcinoma in Southeast Asia [89]. It mostly metastasizes to organs such as liver, lung, and brain via unknown mechanisms [89]. It is characterized by high intra-tumoral lymphocyte infiltrations, expression of EBV-encoded latent genes, including EBERs, EBNA1, LMP1, LMP2, and BARTs, and significant levels of circulating EBV DNA in the plasma of patients [90,91]. Generally, LMP1 is often found in EBV-positive NPCs, and its expression is detected in 20 to 60% of NPC patients [92]. It is considered to be an oncoprotein, due to the presence of COOH-terminal activation regions (CTARs) functional domains. These domains play a crucial role in inflammation and tumorigenesis by interacting with both tumor necrosis factor (TNF) receptor-associated factors and TNF receptor-associated death domain [92].

Most studies have found that tumorigenesis of EBV-associated NPC is strongly related to the LMP1 signaling molecules as well as the activities of p38 MAPKs and JNKs. LMP1, through binding of its CTAR2 to the TNF receptor-associated death domain/TNF receptor-associated factor 2 (TRADD/TRAF2) signaling molecules, can activate the JNK signaling pathway and upregulate AP-1 activity through functional heterodimerization of c-Jun and JunB [93,94]. The heterodimer then binds to the promoter of p16 tumor suppressor gene and downregulates its activity [93]. This causes the reduction of negative control on cyclin D and enables the NPC cells to further progress through the G1/S stage of the cell cycle [93].

On the other hand, activation of the p38 signaling pathway may also increase LMP1 regulation. Both p38α and p38β can induce CREB and ATF1 phosphorylation indirectly. This results in the heterodimerization of the transcription factor ATF1-CREB and activates the LMP1 promoter via a CRE site [95]. Considering that p38 signaling, upon activation by cytokines and environmental stress, can lead to apoptosis, upregulation of LMP1 by the cytokine- or stress-activated p38 may allow EBV-positive cells to escape from apoptosis [95]. A platelet-derived endothelial cell growth factor called thymidine phosphorylase (TP) has been found to be associated with poor prognosis in EBV-associated NPC. The expression level of TP can be induced by activation of the p38 MAPK pathway via the CTAR1 and CTAR2 domains of LMP1 [96]. It is suggested that JNK and p38 MAPK activation by LMP1 might contribute to the development of radio-resistance in NPC, but the detailed molecular mechanism is yet to be understood [97,98]. A study reported that JNK activation by LMP1 in EBV-associated NPC could promote the expression of hypoxia-induced factor 1 (HIF-1) and vascular endothelial growth factor (VEGF), which eventually contributed towards radio-resistance in NPC patients [98]. Moreover, LMP-1 also inhibits an anti-metastatic protein tissue inhibitor of metalloproteinase-3 (TIMP-3) through transcriptional repression via the p38 MAPK pathway to promote metastasis [99].

### 5.4. Activation of p38 MAPK Signaling Promotes Inflammatory Tumor Microenvironment

Chronic inflammation is one of the features of cancer progression caused by the immune cells. It increases the risk of cancer transformation in normal cells, as well as promoting cancer cell metastasis and invasion [100]. NPC has a distinct tumor microenvironment in which the epithelial tumor cells are surrounded by many infiltrating immune cells. However, the immune response often fails to halt cancer progression. One of the reasons for such failure is that the tumor-associated macrophages (TAMs) fail to perform phagocytosis and instead produce signals that suppress the adaptive immune response. For instance, activation of cyclic adenosine monophosphate (cAMP)-dependent protein kinase (PKA) by prostaglandin E2 (PGE2) results in the activation of p38 in TAMs [101]. This causes the stabilization of transcription factor CCAAT/enhancer binding protein d (C/EBPd) mRNA via nucleocytoplasmic shuttling of the RNA binding protein Hu antigen R (HuR) [102]. An increase of C/EBPd abundance in macrophages in response to PGE2 may lead to enhanced production of IL-10 and pentraxin 3 (PTX3), which suppresses the ability of macrophages to phagocytose NPC cells [102].

### 5.5. Activation of JNK and p38 MAPK Signaling Promotes NPC Cell Invasion and Metastasis

Tumor metastasis refers to the spread and implantation of tumor cells from the primary tumor site to distant tissues. Tumor metastasis is generally implicated in disease progression, decreased survival, and reduced response to therapy. There are many steps and requirements for tumor metastasis, including local tumor cell infiltration into neighboring tissue, intravasation (trans-endothelial cancer cell movement into arteries), survival in the circulatory system, extravasation, and subsequent proliferation in competent organs leading to colonization [103]. In this process, matrix metalloproteinases (MMPs), a family of zinc metallo-endopeptidases that is capable of digesting extracellular matrix (ECM) molecules, have been implicated as crucial molecules in the degradation of ECM [104]. In addition, epithelial–mesenchymal transition (EMT) also plays an important role in tumor metastasis, by which the cancer cells need to lose their cell-to-cell adhesion and gain the abilities of invasion and motility to become mesenchymal cells for metastasis [105]. Among them, MMP-2 and MMP-9 are known to facilitate the invasion and metastasis of head and neck carcinoma [106]. An increase in expression of MMP-2, MMP-9, and angiogenic cytokine VEGF in NPC cells has also been found to be closely correlated with high metastatic potential of NPC [107,108]. It is shown that treatment with p38 MAPK inhibitor (SB203580) and transfection with p38 MAPK siRNA can downregulate both MMP-2 and VEGF, which in turn inhibits NPC cell invasion [108]. Downregulation of phosphorylated p38 and phosphorylated JNK1/2 via knockdown of amyloid β precursor protein (APP) exerts inhibitory effects towards EMT in NPC by diminishing the mRNA expression levels of MTA-1, MMP-2, and MMP-9 [109].

### 5.6. Activation of p38 MAPK Signaling Promotes Angiogenesis

Tumor angiogenesis is the process of vasculature formation which supports the growing tumor with sufficient oxygen and nutrients. It is demonstrated to be regulated by the balance of pro-angiogenic [e.g., VEGF, fibroblast growth factor (FGF-2)], platelet-derived growth factor subunit B (PDGFB), and soluble vascular cell adhesion molecule (VCAM)] and anti-angiogenic (e.g., angiostatin and endostatin) factors present in the tumor microenvironment [110]. In NPC tumor tissues, VEGF, as a potent angiogenic factor, is highly expressed, and its expression is correlated with micro-vessel density [111,112]. The p38 MAPK pathway has been proven to be responsible for VEGF-stimulated endothelial cell migration through the regulation of its downstream target genes and proteins, rather than direct p38 signaling [113]. For example, p38α and p38γ activities are required for endothelial cell migration stimulated by both VEGF and non-growth factor stimulants, S1P and VCAM, through the MKK3-p38α/γ-MAPK2 pathway [114]. With these signaling molecules in endothelial cell migration, the p38 MAPK pathway also regulates endogenous urokinase plasminogen activator (uPA) expression, facilitating actin reorganization and focal adhesion assembly [114]. These processes enable the formation of stress fibers which direct cancer cell migration and allow for endothelial cell contraction [115].

## 6. Tumor Suppressive Functions of JNK and p38 MAPK Signaling in NPC

Although JNK and p38 MAPK pathways can directly regulate the aforementioned genes that ultimately support tumor growth and progression, several studies have suggested contradictory functions of JNK and p38 MAPK signaling as positive regulators of tumor suppressor genes.

While many studies have attempted to evaluate the anti-cancer activities of natural compounds in NPC, reports have intriguingly shown that certain natural compounds kill NPC cells via upregulation of JNK and/or p38 MAPK activities. Amongst them, one demonstrated that there was an increase in phosphorylation of p38 and JNK in post-tolfenamic acid therapy that eventually induced NPC cell apoptosis as well as reduction of EMT by downregulation of Slug [116]. Another study reported that caffeic acid phenethyl ester (CAPE) treatment triggered phosphorylation of both JNK and p38 MAPK pathways and reduced EMT by inducing n-myc downstream regulated 1 (NDRG1) expression, a tumor suppressor gene, which in turn led to decreased Slug, Snail, vimentin, and N-cadherin expressions and increased E-cadherin expression [117]. In addition, Liu et al. (2020) also showed that asiatic acid extracted from *Centella asiatica* could induce apoptosis via phosphorylation of p38 MAPK and activation of two pro-apoptotic proteins, BAX and BCL-2 homologous antagonist/killer (BAK), in cisplatin-resistant human NPC-039 and NPC-BM cells [118]. It also triggered the upregulation of caspase-3, caspase-8, caspase-9, and poly (ADP-ribose) polymerase (PARP), which further decreased the NPC cell viability [118]. Treatment with celastrol, a traditional Chinese medicinal plant, was also found to significantly increase the phosphorylation of p38 MAPK and JNK1/2 in cisplatin-resistant human NPC-039 and NPC-BM NPC cells, triggering cytotoxicity by activation of caspase-mediated apoptotic pathways in these cells [119]. Cantharidic acid, a natural toxin secreted by beetles, has proven to reduce NPC cell viability through the upregulation of caspase activation in extrinsic and intrinsic apoptosis pathways, as well as the upregulation of p38 and JNK1/2 pathways [120]. Treatment with licochalcone A significantly promoted cell apoptosis by increasing p38 and JNK1/2 activation and upregulated caspase-3, caspase-8, caspase-9 activation, and cleaved-PARP expression [121]. Similarly, a phenol compound, Hispolon, isolated from *Phellinus linteus* inhibited cell proliferation of HONE-1 and NP-039 cell lines by activating caspases activation and PARP cleavage via p38 MAPK and JNK1/2 pathways [122].

Together with the evidence mentioned above, it is hypothesized that the anti-tumor effects might be triggered by activating p38 MAPK and JNK pathways. However, none attempted to determine how p38 MAPK and JNK pathways could have such tumor suppressive functions in NPC cells. Indeed, the precise mechanisms for how p38 MAPKs and JNKs could act as tumor suppressors in NPC and the cellular contexts that govern the pro- and anti-cancer activities of p38 MAPKs and JNKs have yet to be ascertained and warrant further investigations.

## 7. Targeting JNK and p38 MAPK in NPC

With the important roles of JNKs and p38 MAPKs in NPC pathogenesis, targeting JNK and p38 MAPK might be a promising strategy to be considered in NPC treatment. To date, some potent and specific JNK and p38 MAPK inhibitors have been developed, and both of their in vitro and in vivo activities are summarized in Table 3 and Table 4, respectively. Some of the inhibitors are currently being tested in clinical trials for other diseases [123,124,125,126,127,128,129]. One of the p38 MAPK inhibitors, ralimetinib (LY2228820), has entered phase I and phase II clinical trials in patients with advanced cancer [130].

SP600125 (JNK inhibitor) and SB203580 (p38 MAPK inhibitor) are the two most common MAPK inhibitors used in NPC studies. Combinations of SB203580 with polyphyllin G resulted in a decrease in cell viability and increase in autophagy [155]. Likewise, EBV-positive NPC cells that underwent treatment with SP600125 were more sensitive to radiation compared to the untreated cells [98]. However, some studies have reported the opposite effects. Pre-treatments by SP600125 and SB203580 partly inhibited the CAPE effect on NDRG1 induction in NPC cells by inhibiting phosphorylation of JNK or p38 and CAPE-induced NDRG1 protein levels [117]. Combination of licochalcone A with SB203580 treatment significantly diminished the licochalcone A-induced cleavage of caspase-9, caspase-8, and caspase-3, which would abolish the induction of apoptosis by licochalcone A monotherapy [121].

At present, there are still insufficient evidence and studies about the combination of chemotherapy with the other JNK and p38 MAPK inhibitors to make conclusions of their combinatory effects for NPC treatment. Extreme suppression of total JNK and p38 MAPK activities should be practiced with caution, as different isoforms have distinct functions in cell context-dependent manners. For instance, the development of SP600125 towards clinical trials was terminated due to lack of selectivity towards the target site of cancer cells [128]. No clinical trial has been initiated to test the efficiency of JNK and p38 MAPK inhibitors towards NPC.

## 8. Conclusions

The activities of JNKs and p38 MAPKs in NPC cell survival, invasion and progression, and inflammatory response may be critical determinants of tumor cell survival and metastasis. Intriguingly, these signaling pathways might be either oncogenic or occasionally tumor suppressive through mechanisms which have yet to be fully elucidated in NPC. It is likely that their functions might be conditional on whether the activation of JNKs and p38 MAPKs is transient or prolonged, or even differential by the EBV status of NPC [156]. Nevertheless, more studies are needed to dissect the regulation and functions of different JNKs and p38 MAPKs in NPC, particularly in consideration of the extensive crosstalk and integration between JNK and p38 MAPK signaling pathways in the context of tumor growth and progression. Understanding these pathways will be of fundamental importance to justify the use of JNK and p38 MAPK inhibitors in NPC and to design better combinatorial treatment strategies in NPC. Moreover, whether any p38 MAPKs and JNKs can be exploited as diagnostic and predictive biomarkers for NPC patients also warrants further exploration.

## Figures and Tables

**Table 1:** Status of JNKs in human cancers and their clinical implications.

Type of Cancer	JNK Status	Clinical Implications	References
**Liver cancer**	JNK1 activity upregulated	Higher expression of JNK1, rather than JNK2, was detected as a progenitor cell biomarker and lowered the survival rate of patients with hepatocellular carcinoma (HCC).	[46]
**Prostate cancer**	JNK1,2 activities upregulated in silico	Total JNK expression was upregulated in human malignant prostate epithelium compared to normal or benign hyperplasic (BPH) epithelium.	[47,48]
**Breast cancer**	JNK1,2 activities downregulated	Decreased p-JNK1/2 expression was observed in breast infiltrating ductal carcinoma (IDC) cases and was correlated significantly with increased tumor grade and decreased age at diagnosis.	[49]
**Bladder cancer**	JNK2 activity downregulated	Lower JNK2 expression was associated with poorer overall survival among patients who underwent radical cystectomy.	[50]
**Lung cancer**	JNK1,2 activity downregulated	JNK1/2 was inactivated in human lung squamous cell carcinoma (LSCC) and their activities were positively correlated with survival rates of patients.	[51]
**Thyroid cancer**	JNK activity upregulated	p-JNK was overexpressed in papillary thyroid carcinomas and was significantly associated with the presence of lymph node metastases and advanced TNM stages.	[52]
**Colorectal cancer**	JNK1 activity upregulated	JNK activity was elevated in human colorectal tumors compared to normal intestinal mucosa. p-JNK1 was overexpressed in the multidrug-resistant colon cancer cells.	[53]
**Head and neck squamous cell carcinoma**	JNK1,2 activities downregulated	Higher JNK1/2 activities had better survival rate than those with lower JNK1/2 activities in patients with head and neck squamous cell carcinoma tumors.	[51,54]
**Skin cancer**	JNK1 activity downregulated, JNK2 activity upregulated	JNK1 expression was inhibited by squamous cell carcinoma antigen (SCCA) and blocked UV-induced keratinocyte apoptosis. JNK2 was activated in more than 70% of human squamous cell carcinoma (SCC) and is sufficient to couple with oncogenic Ras to transform primary human epidermal cells into malignancy.	[55,56]
**Ovarian cancer**	JNK1 activities upregulated	JNK1 expression levels were found to be higher in advanced stage (III and IV) cases than in early stage (I and II) cases and inversely associated with the survival of ovarian cancer patients.	[57,58]
**Nasopharyngeal carcinoma**	JNK activity upregulated	Activation of JNK signaling was associated with TNM staging of NPC, as NPC patients with stage III–IV had higher positive expression rates of JNK and p-JNK proteins compared to NPC patients with stage I–II.	[59]

**Table 2:** Status of p38 MAPKs in human cancers and their clinical implications.

Type of Cancer	p38 MAPK Status	Clinical Implications	References
Liver cancer	p38γ, δ activities upregulated	High p38γ expression was associated with a poorer outcome in cases of liver cancer. Overexpression of p38δ was observed in cholangiocarcinoma and responsible for cancer cell motility and invasion.	[60,61]
Prostate cancer	p38 MAPKs upregulated	Strong expression of p38 MAPKs was observed in all prostate cancer patients with progressive disease from stages II to IV.	[62]
Breast cancer	p38α, δ activities upregulated	High levels of active p38α were correlated with poor prognosis, lymph node metastasis, and tamoxifen resistance in breast cancer patients. High p38δ levels were associated with poor prognosis in breast cancer patients of all tumor subtypes, especially estrogen receptor (ER)-positive/human epidermal growth factor receptor 2 (HER2)-negative types.	[63,64,65]
Bladder cancer	p38 activity upregulated	The expression of p38 in transitional cell carcinoma (TCC) of the bladder was positively correlated with depth of muscle invasion, grade, stage, lymph node metastasis, distant metastasis, size, and number of tumors.	[66,67]
Lung cancer	p38α activity upregulated	Higher numbers of both phosphorylated-p38 and p38α-positive cells were observed in lung adenocarcinoma compared to the normal lung parenchyma and correlated with a higher mortality rate as well as with a shorter time to relapse.	[68,69]
Thyroid cancer	p38α activity upregulated	High expression of p38α was revealed in malignant thyroid carcinoma, such as human papillary and follicular thyroid carcinomas.	[70]
Colorectal cancer	p38α, β, δ activities upregulated	High levels of phosphorylated p38, p38α, and p38β were correlated with chemotherapy resistance and poor overall survival in colon cancer patients. The depletion of p38δ impaired tumor growth in vivo.	[71,72,73]
Head and neck squamous cell carcinoma	p38α, δ activities upregulated	Expression of p38α and p38δ by tumor cells was detected in HNSCCs in vivo. Phosphorylated p38 expression was clearly increased in moderately differentiated and even further increased in poorly differentiated HNSCC, with increased angiogenesis and lymph angiogenesis.	[74,75]
Skin cancer	p38α, δ activities upregulated	Increased expression levels of p38α and p38δ were detected in human primary cutaneous SCCs.	[76]
Nasopharyngeal carcinoma	p38 MAPKs upregulated	p38 MAPKs were overexpressed in non-keratinizing squamous cell carcinoma (most common form in high-risk countries) at T3–T4, N2–N3 and clinical stage III–IV.	[77,78]

**Table 3:** Examples and specificity of JNK inhibitors as well as their potential in cancers.

Name	IC50 (nM)				Potential Usages in Cancer	References
	JNK1	JNK2	JNK3	JNKs		
SP600125	40	40	90	ND	Anti-cancer effects in stomach cancer, oral squamous carcinoma, lung adenocarcinoma, cholangiocarcinoma, colon carcinoma, pancreatic cancer, and glioblastoma.	[131,132,133,134,135,136,137,138]
JNK-IN-1	ND	ND	ND	2.31	Anti-cancer effects in skin cancer.	[139]
JNK-IN-8	4.67	18.7	0.98	ND	Sensitized triple-negative breast cancer cells to lapatinib.	[140,141]
Bentamapimod (AS602801/PGL5001)	80	90	230	ND	Induced apoptosis of cancer stem cells.	[142]
BI-78D3	ND	ND	ND	280	Anti-cancer effects in osteosarcoma.	[143]
CC-401	25–50	500–1000	25–50	ND	Anti-cancer effects in colon cancer and acute myeloid leukemia.	[128,144]

ND: Not determined.

**Table 4:** Examples and specificity of p38 MAPK inhibitors as well as their potential in cancers.

Name	IC50 (nM)					Potential Usages in Cancer	References
	p38 MAPKs	p38α	p38β	p38γ	p38δ		
SB203580	50	ND	500	ND	ND	Anti-cancer effects in breast cancer.	[145]
Doramapimod (BIRB 796)	0.1	38	65	520	200	Anti-cancer effects in multiple myeloma, oral epidermoid carcinoma, cervical cancer.	[146,147,148]
Talmapimod (SCIO-469)	ND	9	90	ND	ND	Potential chemotherapy for multiple myeloma and leukemia.	[149,150]
Ralimetinib (LY2228820)	ND	5.3	3.2	ND	ND	Potential chemotherapy for melanoma, non-small cell lung cancer, ovarian cancer, glioma, myeloma, breast cancer, colorectal cancer, sarcoma, renal cancer, and pancreatic cancer.	[130,151]
Losmapimod (GW856553X)	ND	8.1	7.6	ND	ND	Overcame gefitinib resistance in non-small cell lung cancer (NSCLC).	[152]
Pexmetinib (ARRY-614)	1	35	26	ND	ND	Potential chemotherapy for hematological carcinoma, such as myelodysplastic syndromes.	[153,154]

ND: Not determined.

## Data Availability

Not applicable.

## References

[1] Bruce J.P., Yip K., Bratman S.V., Ito E., Liu F. (2015). Nasopharyngeal cancer: Molecular landscape. J. Clin. Oncol..

[2] Chen Y., Chan A.T., Le Q., Blanchard P., Sun Y., Ma J. (2019). Nasopharyngeal carcinoma. Lancet.

[3] Zhao L., Fong A.H., Liu N., Cho W.C. (2018). Molecular subtyping of nasopharyngeal carcinoma (NPC) and a microRNA-based prognostic model for distant metastasis. J. Biomed. Sci..

[4] American Cancer Society (2017). Key Statistics for Nasopharyngeal Cancer. https://www.cancer.org/cancer/nasopharyngeal-cancer/about/key-statistics.html.

[5] Young L.S., Dawson C.W. (2014). Epstein-Barr virus and nasopharyngeal carcinoma. Chin. J. Cancer.

[6] Huang T., Ploner A., Chang E.T., Liu Q., Cai Y., Zhang Z., Chen G., Huang Q., Xie S., Cao S. (2021). Dietary patterns and risk of nasopharyngeal carcinoma: A population-based case-control study in southern China. Am. J. Clin. Nutr..

[7] Lin Z., Khong B., Kwok S., Cao H., West R.B., Le Q., Kong C.S. (2014). Human papillomavirus 16 detected in nasopharyngeal carcinomas in white Americans but not in endemic Southern Chinese patients. Head Neck.

[8] Tsao S.W., Yip Y.L., Tsang C.M., Pang P.S., Lau V.M.Y., Zhang G., Lo K.W. (2014). Etiological factors of nasopharyngeal carcinoma. Oral Oncol..

[9] Li J., Lu T., Huang Y., Han F., Chen C., Xiao W. (2010). Clinical features of 337 patients with recurrent nasopharyngeal carcinoma. Chin. J. Cancer.

[10] Lee A.W., Ng W.T., Chan J.Y., Corry J., Mäkitie A., Mendenhall W.M., Rinaldo A., Rodrigo J.P., Saba N.F., Strojan P. (2019). Management of locally recurrent nasopharyngeal carcinoma. Cancer Treat. Rev..

[11] Abdullah B., Alias A., Hassan S. (2009). Challenges in the management of nasopharyngeal carcinoma: A review. Malays J. Med. Sci..

[12] Plotnikov A., Flores K., Maik-Rachline G., Zehorai E., Kapri-Pardes E., Berti D.A., Hanoch T., Besser M.J., Seger R. (2015). The nuclear translocation of ERK1/2 as an anticancer target. Nat. Commun..

[13] Koul H.K., Pal M., Koul S. (2013). Role of p38 MAP kinase signal transduction in solid tumors. Genes Cancer.

[14] Burotto M., Chiou V.L., Lee J., Kohn E.C. (2014). The MAPK pathway across different malignancies: A new perspective. Cancer.

[15] Zhang W., Liu H.T. (2002). MAPK signal pathways in the regulation of cell proliferation in mammalian cells. Cell Res..

[16] Paton E.L., Turner J.A., Schlaepfer I.R. (2020). Overcoming resistance to therapies targeting the MAPK pathway in BRAF-mutated tumours. J. Oncol..

[17] Yadav V., Zhang X., Liu J., Estrem S., Li S., Gong X.Q., Buchanan S., Henry J.R., Starling J.J., Peng S.B. (2012). Reactivation of mitogen-activated protein kinase (MAPK) pathway by FGF receptor 3 (FGFR3)/Ras mediates resistance to vemurafenib in human BRAF V600E mutant melanoma. J. Biol. Chem..

[18] Vo U., Vajpai N., Flavell L., Bobby R., Breeze A.L., Embrey K.J., Golovanov A.P. (2016). Monitoring Ras interactions with the nucleotide exchange factor Son of Sevenless (SOS) using site-specific NMR reporter signals and intrinsic fluorescence. J. Biol. Chem..

[19] Plotnikov A., Zehorai E., Procaccia S., Seger R. (2011). The MAPK cascades: Signaling components, nuclear roles and mechanisms of nuclear translocation. Biochim. Biophys. Acta (BBA)-Mol. Cell Res..

[20] Yu Y., Richardson D.R. (2011). Cellular iron depletion stimulates the JNK and p38 MAPK signaling transduction pathways, dissociation of ASK1-thioredoxin, and activation of ASK. J. Biol. Chem..

[21] Braicu C., Buse M., Busuioc C., Drula R., Gulei D., Raduly L., Rusu A., Irimie A., Atanasov A.G., Slaby O. (2019). A comprehensive review on MAPK: A promising therapeutic target in cancer. Cancers.

[22] Tulalamba W., Janvilisri T. (2012). Nasopharyngeal carcinoma signaling pathway: An update on molecular biomarkers. Int. J. Cell Biol..

[23] Sabapathy K. (2012). Role of the JNK pathway in human diseases. Prog. Mol. Biol. Transl. Sci..

[24] Yarza R., Vela S., Solas M., Ramirez M.J. (2016). c-Jun N-terminal kinase (JNK) signaling as a therapeutic target for Alzheimer’s disease. Front. Pharmacol..

[25] Bogoyevitch M.A., Kobe B. (2006). Uses for JNK: The many and varied substrates of the c-Jun N-terminal kinases. Microbiol. Mol. Biol. Rev..

[26] Gkouveris I., Nikitakis N.G. (2017). Role of JNK signaling in oral cancer: A mini review. Tumor. Biol..

[27] Bubici C., Papa S. (2014). JNK signalling in cancer: In need of new, smarter therapeutic targets. Br. J. Pharm..

[28] Haeusgen W., Boehm R., Zhao Y., Herdegen T., Waetzig V. (2009). Specific activities of individual c-Jun N-terminal kinases in the brain. Neuroscience.

[29] New L., Han J. (1998). The p38 MAP kinase pathway and its biological function. Trends Cardiovasc. Med..

[30] Roy S., Roy S., Rana A., Akhter Y., Hande M.P., Banerjee B. (2018). The role of p38 MAPK pathway in p53 compromised state and telomere mediated DNA damage response. Mutat. Res. Genet. Toxicol. Environ. Mutagenesis.

[31] Yang Y., Kim S.C., Yu T., Yi Y.S., Rhee M.H., Sung G.H., Yoo B.C., Cho J.Y. (2014). Functional roles of p38 mitogen-activated protein kinase in macrophage-mediated inflammatory responses. Mediat. Inflamm..

[32] Whitmarsh A., Davis R. (2007). Role of mitogen-activated protein kinase kinase 4 in cancer. Oncogene.

[33] Cuadrado A., Nebreda A.R. (2010). Mechanisms and functions of p38 MAPK signalling. Biochem. J..

[34] Kyriakis J.M., Avruch J. (2001). Mammalian mitogen-activated protein kinase signal transduction pathways activated by stress and inflammation. Physiol. Rev..

[35] Shiryaev A., Dumitriu G., Moens U. (2011). Distinct roles of MK2 and MK5 in cAMP/PKA-and stress/p38 MAPK-induced heat shock protein 27 phosphorylation. J. Mol. Signal..

[36] Katopodis P., Kerslake R., Zikopoulos A., Beri N.E., Anikin V. (2020). p38β-MAPK11 and its role in female cancers. J. Ovarian Res..

[37] Xu W., Liu R., Dai Y., Hong S., Dong H., Wang H. (2021). The role of p38γ in cancer: From review to outlook. Int. J. Biol. Sci..

[38] O’Callaghan C., Fanning L.J., Barry O.P. (2014). p38δ MAPK: Emerging roles of a neglected isoform. Int. J. Cell Biol..

[39] Jing L., Anning L. (2005). Role of JNK activation in apoptosis: A double-edged sword. Cell Res..

[40] Dhillon A.S., Hagan S., Rath O., Kolch W. (2007). MAP kinase signalling pathways in cancer. Oncogene.

[41] Wang J., Tai G. (2016). Role of c-Jun N-terminal kinase in hepatocellular carcinoma development. Target. Oncol..

[42] Feng C., He K., Zhang C., Su S., Li B., Li Y., Duan C.Y., Chen S., Chen R., Liu Y. (2014). JNK contributes to the tumorigenic potential of human cholangiocarcinoma cells through the mTOR pathway regulated GRP78 induction. PLoS ONE.

[43] Bhowmick N.A., Zent R., Ghiassi M., McDonnell M., Moses H.L. (2001). Integrin beta 1 signaling is necessary for transforming growth factor-beta activation of p38 MAPK and epithelial plasticity. J. Biol. Chem..

[44] Cheng T., Symons M., Jou T. (2004). Regulation of anoikis by Cdc42 and Rac. Exp. Cell Res..

[45] Martínez-Limón A., Joaquin M., Caballero M., Posas F., De Nadal E. (2020). The p38 pathway: From biology to cancer therapy. Int. J. Mol. Sci..

[46] Chang Q., Chen J., Beezhold K.J., Castranova V., Shi X., Chen F. (2009). JNK1 activation predicts the prognostic outcome of the human hepatocellular carcinoma. Mol. Cancer.

[47] Xu R., Hu J. (2020). The role of JNK in prostate cancer progression and therapeutic strategies. Biomed. Pharmacother..

[48] Royuela M., Arenas M.I., Bethencourt F.R., Sánchez-Chapado M., Fraile B., Paniagua R. (2002). Regulation of proliferation/apoptosis equilibrium by mitogen-activated protein kinases in normal, hyperplastic, and carcinomatous human prostate. Hum. Pathol..

[49] Yeh Y., Hou M., Chung Y., Chen Y., Yang S., Chen D., Su J., Yuan S.F. (2006). Decreased expression of phosphorylated JNK in breast infiltrating ductal carcinoma is associated with a better overall survival. Int. J. Cancer.

[50] Pan C.W., Liu H., Zhao Y., Qian C., Wang L., Qi J. (2016). JNK2 downregulation promotes tumorigenesis and chemoresistance by decreasing p53 stability in bladder cancer. Oncotarget.

[51] Liu J., Wang T., Creighton C.J., Wu S., Ray M., Janardhan K.S., Willson C.J., Cho S., Castro P.D., Ittmann M.M. (2019). JNK 1/2 represses Lkb 1-deficiency-induced lung squamous cell carcinoma progression. Nat. Commun..

[52] Wang X., Chao L., Zhen J., Chen L., Ma G., Li X. (2010). Phosphorylated c-Jun NH2-terminal kinase is overexpressed in human papillary thyroid carcinomas and associates with lymph node metastasis. Cancer Lett..

[53] Zhu M.M., Tong J.L., Xu Q., Nie F., Xu X.T., Xiao S.D., Ran Z.H. (2012). Increased JNK1 signaling pathway is responsible for ABCG2-mediated multidrug resistance in human colon cancer. PLoS ONE.

[54] Gkouveris I., Nikitakis N., Karanikou M., Rassidakis G., Sklavounou A. (2016). JNK1/2 expression and modulation of STAT3 signaling in oral cancer. Oncol. Lett..

[55] Hammouda M.B., Ford A.E., Liu Y., Zhang J.Y. (2020). The JNK signaling pathway in inflammatory skin disorders and cancer. Cells.

[56] Katagiri C., Nakanishi J., Kadoya K., Hibino T. (2006). Serpin squamous cell carcinoma antigen inhibits UV-induced apoptosis via suppression of c-JUN NH2-terminal kinase. J. Cell Biol..

[57] Rodriguez-Aguayo C., Vivas-Mejia P., Benito J.M., Fernandez A., Francois-Xavier C., Chavez-Reyes A., Sood A.K., Lopez-Berestein G. (2010). JNK-1 inhibition leads to antitumor activity in ovarian cancer. Clin. Cancer Res..

[58] Seino M., Okada M., Sakaki H., Takeda H., Watarai H., Suzuki S., Seino S., Kuramoto K., Ohta T., Nagase S. (2016). Time-staggered inhibition of JNK effectively sensitizes chemoresistant ovarian cancer cells to cisplatin and paclitaxel. Oncol. Rep..

[59] Zhang Y., Zhang M., Zhou H., Yang J. (2018). Activation of c-Jun/JNK signaling predicts poor prognosis in nasopharyngeal carcinoma. Int. J. Clin. Exp. Pathol..

[60] Tomás-Loba A., Manieri E., González-Terán B., Mora A., Leiva-Vega L., Santamans A.M., Romero-Becerra R., Rodríguez E., Pintor-Chocano A., Feixas F. (2019). p38γ is essential for cell cycle progression and liver tumorigenesis. Nature.

[61] Tan F.L., Ooi A., Huang D., Wong J.C., Qian C., Chao C., Ooi L., Tan Y., Chung A., Cheow P. (2010). p38 delta/MAPK13 as a diagnostic marker for cholangiocarcinoma and its involvement in cell motility and invasion. Int. J. Cancer.

[62] Browne A., Göbel A., Thiele S., Hofbauer L., Rauner M., Rachner T. (2016). p38 MAPK regulates the Wnt inhibitor Dickkopf-1 in osteotropic prostate cancer cells. Cell Death Dis..

[63] Suarez-Cuervo C., Merrell M.A., Watson L., Harris K.W., Rosenthal E.L., Väänänen H.K., Selander K.S. (2004). Breast cancer cells with inhibition of p38α have decreased MMP-9 activity and exhibit decreased bone metastasis in mice. Clin. Exp. Metastasis.

[64] Wada M., Canals D., Adada M., Coant N., Salama M.F., Helke K.L., Arthur J., Shroyer K.R., Kitatani K., Obeid L.M. (2017). p38 delta MAPK promotes breast cancer progression and lung metastasis by enhancing cell proliferation and cell detachment. Oncogene.

[65] Wagner E.F., Nebreda Á.R. (2009). Signal integration by JNK and p38 MAPK pathways in cancer development. Nat. Rev. Cancer.

[66] Harb O.A., Haggag R., Ali M.M., El Shorbagy S., Abdelbary A.M., Abdelaziz L.A., Salim R.A., Abdel Wahab K.M. (2017). The prognostic role of NEDD9 and p38 protein expression levels in urinary bladder transitional cell carcinoma. J. Oncol..

[67] Kumar B., Koul S., Petersen J., Khandrika L., Hwa J.S., Meacham R.B., Wilson S., Koul H.K. (2010). p38 mitogen-activated protein kinase-driven MAPKAPK2 regulates invasion of bladder cancer by modulation of MMP-2 and MMP-9 activity. Cancer Res..

[68] Greenberg A.K., Basu S., Hu J., Yie T., Tchou-Wong K.M., Rom W.N., Lee T.C. (2002). Selective p38 activation in human non-small cell lung cancer. Am. J. Respir. Cell Mol. Biol..

[69] Vitos-Faleato J., Real S.M., Gutierrez-Prat N., Villanueva A., Llonch E., Drosten M., Barbacid M., Nebreda A.R. (2020). Requirement for epithelial p38 alpha in KRAS-driven lung tumor progression. Proc. Natl. Acad. Sci. USA.

[70] Pomerance M., Quillard J., Chantoux F., Young J., Blondeau J. (2006). High-level expression, activation, and subcellular localization of p38-MAP kinase in thyroid neoplasms. J. Pathol..

[71] Stramucci L., Pranteda A., Stravato A., Amoreo C.A., Pennetti A., Diodoro M.G., Bartolazzi A., Milella M., Bossi G. (2019). MKK3 sustains cell proliferation and survival through p38 delta MAPK activation in colorectal cancer. Cell Death Dis..

[72] Paillas S., Boissiere F., Bibeau F., Denouel A., Mollevi C., Causse A., Denis V., Vezzio-Vie N., Marzi L., Cortijo C. (2011). Targeting the p38 MAPK pathway inhibits irinotecan resistance in colon adenocarcinoma. Cancer Res..

[73] Fan X., Wan X., Fu X., Wu P., Chen D., Wang P., Jiang L., Wang D., Chen Z., Huang Y. (2014). Phosphorylated p38, a negative prognostic biomarker, complements TNM staging prognostication in colorectal cancer. Tumor. Biol..

[74] Junttila M., Ala-Aho R., Jokilehto T., Peltonen J., Kallajoki M., Grenman R., Jaakkola P., Westermarck J., Kähäri V. (2007). p38α and p38δ mitogen-activated protein kinase isoforms regulate invasion and growth of head and neck squamous carcinoma cells. Oncogene.

[75] Leelahavanichkul K., Amornphimoltham P., Molinolo A.A., Basile J.R., Koontongkaew S., Gutkind J.S. (2014). A role for p38 MAPK in head and neck cancer cell growth and tumor-induced angiogenesis and lymphangiogenesis. Mol. Oncol..

[76] Haider A.S., Peters S.B., Kaporis H., Cardinale I., Fei J., Ott J., Blumenberg M., Bowcock A.M., Krueger J.G., Carucci J.A. (2006). Genomic analysis defines a cancer-specific gene expression signature for human squamous cell carcinoma and distinguishes malignant hyperproliferation from benign hyperplasia. J. Investig. Derm..

[77] Asnir R., Yudhistira A., Daulay E., Muzakkir M., Yulius S. (2018). p38 mitogen-activated protein kinase (p38 MAPK) overexpression in clinical staging of nasopharyngeal carcinoma. IOP Conference Series: Earth and Environmental Sciencep38 Mitogen-Activated Protein Kinase (p38 MAPK) Overexpression in Clinical Staging of Nasopharyngeal Carcinoma.

[78] Farhat F., Daulay E.R., Chrestella J., Asnir R.A., Yudhistira A., Susilo R.R. (2018). Correlation of p38 mitogen-activated protein kinase expression to clinical stage in nasopharyngeal carcinoma. Open Access Maced. J. Med. Sci..

[79] Sun Y., Chen K., Lin G., Wan F., Chen L., Zhu X. (2021). Silencing c-Jun inhibits autophagy and abrogates radioresistance in nasopharyngeal carcinoma by activating the PI3K/AKT/mTOR pathway. Ann. Transl. Med..

[80] Huang C., Wang J., Kikkawa U., Mukai H., Shen M., Morita I., Chen B., Chang W. (2008). Calcineurin-mediated dephosphorylation of c-Jun Ser-243 is required for c-Jun protein stability and cell transformation. Oncogene.

[81] Zuo Q., Shi M., Chen J., Liao W. (2011). The Ras signaling pathway mediates cetuximab resistance in nasopharyngeal carcinoma. Biomed. Pharmacother..

[82] Lu J., Luo H., Liu X., Peng Y., Zhang B., Wang L., Xu X., Peng X., Li G., Tian W. (2014). miR-9 targets CXCR4 and functions as a potential tumor suppressor in nasopharyngeal carcinoma. Carcinogenesis.

[83] Vatte C., Al Amri A.M., Cyrus C., Chathoth S., Ahmad A., Alsayyah A., Ali A.A. (2021). Epstein-Barr virus infection mediated TP53 and BCL2 expression in nasopharyngeal carcinoma pathogenesis. Mol. Clin. Oncol..

[84] Fendri A., Kontos C.K., Khabir A., Mokdad-Gargouri R., Scorilas A. (2011). BCL2L12 is a novel biomarker for the prediction of short-term relapse in nasopharyngeal carcinoma. Mol. Med..

[85] Chen M., Yang S., Lai J., Yeh K., Yang J., Chen L., Chen H. (2010). Expression of BCL2 correlates with poor prognosis and modulates migration of nasopharyngeal carcinoma cells. Clin. Chim. Acta.

[86] Zhen Y., Liu Z., Yang H., Yu X., Wu Q., Hua S., Long X., Jiang Q., Song Y., Cheng C. (2013). Tumor suppressor PDCD4 modulates miR-184-mediated direct suppression of c-Myc and BCL2 blocking cell growth and survival in nasopharyngeal carcinoma. Cell Death Dis..

[87] Song Q., Wang G., Chu Y., Zhou L., Jiang M., He Q., Liu M., Qin J., Hu J. (2013). TNF-alpha up-regulates cellular inhibitor of apoptosis protein 2 (c-IAP2) via c-Jun N-terminal kinase (JNK) pathway in nasopharyngeal carcinoma. Int. Immunopharmacol..

[88] Xie M., Yi X., Wang R., Wang L., He G., Zhu M., Qi C., Liu Y., Ye Y., Tan S. (2014). 14-Thienyl methylene matrine (YYJ18), the derivative from matrine, induces apoptosis of human nasopharyngeal carcinoma cells by targeting MAPK and PI3K/Akt pathways in vitro. Cell Physiol. Biochem..

[89] Ngan H., Wang L., Lo K., Lui V.W.Y. (2018). Genomic landscapes of EBV-associated nasopharyngeal carcinoma vs. HPV-associated head and neck cancer. Cancers.

[90] Tsao S.W., Tsang C.M., Lo K.W. (2017). Epstein-Barr virus infection and nasopharyngeal carcinoma. Philos. Trans. R. Soc. B Biol. Sci..

[91] Cao Y., Xie L., Shi F., Tang M., Li Y., Hu J., Zhao L., Zhao L., Yu X., Luo X. (2021). Targeting the signaling in Epstein-Barr virus-associated diseases: Mechanism, regulation, and clinical study. Signal Transduct. Target. Ther..

[92] Lo A.K., Dawson C.W., Lung H.L., Wong K., Young L.S. (2021). The role of EBV-encoded LMP1 in the NPC tumour microenvironment: From function to therapy. Front. Oncol..

[93] Song X., Ai M., Chen X., Deng X., Tao Y., Gong J., Wu Q., Cao Y. (2004). Regulation of c-Jun/JunB heterodimers mediated by Epstein-Barr virus encoded latent membrane protein 1 on Pchinese. Sci. Bull..

[94] Hu Z., Zeng L., Tao Y.G., Tang F.Q., Wang H., Luo F.J., Yi W., Cao Y. (2002). EB virus-encoded latent membrane protein 1 activates the JNK signalling pathway via a mechanism involving TRADD and TRAF in nasopharyngeal carcinoma cell. Prog. Biochem. Biophys..

[95] Johansson P., Jansson A., Ruetschi U., Rymo L. (2010). The p38 signaling pathway upregulates expression of the Epstein-Barr virus LMP1 oncogene. J. Virol..

[96] Chen C., Chen L., Liang Y., Tsang N., Chang Y. (2010). Epstein-Barr virus latent membrane protein 1 induces the chemotherapeutic target, thymidine phosphorylase, via NF-kB and p38 MAPK pathways. Cell. Signal..

[97] Zhang Z., Yu X., Zhou Z., Li B., Peng J., Wu X., Luo X., Yang L. (2019). LMP1-positive extracellular vesicles promote radioresistance in nasopharyngeal carcinoma cells through p38 MAPK signaling. Cancer Med..

[98] Yang L., Liu L., Xu Z., Liao W., Feng D., Dong X., Xu S., Xiao L., Lu J., Luo X. (2015). EBV-LMP1 targeted DNAzyme enhances radiosensitivity by inhibiting tumor angiogenesis via the JNKs/HIF-1 pathway in nasopharyngeal carcinoma. Oncotarget.

[99] Chang S., Chang H., Hung W. (2008). Transcriptional repression of tissue inhibitor of metalloproteinase-3 by Epstein-Barr virus latent membrane protein 1 enhances invasiveness of nasopharyngeal carcinoma cells. Oral Oncol..

[100] Greten F.R., Grivennikov S.I. (2019). Inflammation and cancer: Triggers, mechanisms, and consequences. Immunity.

[101] Al-Taei S., Salimu J., Spary L.K., Clayton A., Lester J.F., Tabi Z. (2017). Prostaglandin E2-mediated adenosinergic effects on CD14 cells: Self-amplifying immunosuppression in cancer. Oncoimmunology.

[102] Hsiao Y.W., Li C.F., Chi J.Y., Tseng J.T., Chang Y., Hsu L.J., Lee C.H., Chang T.H., Wang S.M., Wang D.D. (2013). CCAAT/enhancer binding protein delta in macrophages contributes to immunosuppression and inhibits phagocytosis in nasopharyngeal carcinoma. Sci. Signal.

[103] Yang J., Antin P., Berx G., Blanpain C., Brabletz T., Bronner M., Campbell K., Cano A., Casanova J., Christofori G. (2020). Guidelines and definitions for research on epithelial-mesenchymal transition. Nat. Rev. Mol. Cell Biol..

[104] Singh D., Srivastava S.K., Chaudhuri T.K., Upadhyay G. (2015). Multifaceted role of matrix metalloproteinases (MMPs). Front. Mol. Biosci..

[105] Ribatti D., Tamma R., Annese T. (2020). Epithelial-mesenchymal transition in cancer: A historical overview. Transl. Oncol..

[106] Roomi M.W., Bhanap B., Niedzwiecki A., Rath M. (2017). In vitro modulation of MMP-2 and MMP-9 secretion by cytokines, inducers, and inhibitors in head and neck squamous carcinoma cells (FaDu) and tongue carcinoma cells (SCC-25). J. Otolaryngol. Rhinol..

[107] Wong T., Kwong D., Sham J., Wei W., Kwong Y., Yuen A. (2004). Clinicopathologic significance of plasma matrix metalloproteinase-2 and-9 levels in patients with undifferentiated nasopharyngeal carcinoma. Eur. J. Surg. Oncol..

[108] Lin M., Lu Y., Chung J., Wang S., Lin H., Kang S., Tang C., Ko J., Chen S. (2010). Down-regulation of MMP-2 through the p38 MAPK-NF-κB-dependent pathway by aloe-emodin leads to inhibition of nasopharyngeal carcinoma cell invasion. Mol. Carcinog..

[109] Xu J., Ying Y., Xiong G., Lai L., Wang Q., Yang Y. (2019). Amyloid β precursor protein silencing attenuates epithelial-mesenchymal transition of nasopharyngeal carcinoma cells via inhibition of the MAPK pathway. Mol. Med. Rep..

[110] Ghosh Dastidar D., Ghosh D., Chakrabarti G. (2020). Tumour vasculature targeted anti-cancer therapy. Vessel Plus.

[111] Rivera-Soto R., Damania B. (2019). Modulation of angiogenic processes by the human gammaherpesviruses, Epstein-Barr virus and Kaposi’s sarcoma-associated herpesvirus. Front. Microbiol..

[112] Cheng J., Chen J., Xue K., Wang Z., Yu D. (2018). Clinicopathologic and prognostic significance of VEGF, JAK2 and STAT3 in patients with nasopharyngeal carcinoma. Cancer Cell Int..

[113] Yoshizuka N., Chen R.M., Xu Z., Liao R., Hong L., Hu W., Yu G., Han J., Chen L., Sun P. (2012). A novel function of p38-regulated/activated kinase in endothelial cell migration and tumor angiogenesis. Mol. Cell Biol..

[114] Yu J., Bian D., Mahanivong C., Cheng R.K., Zhou W., Huang S. (2004). p38 mitogen-activated protein kinase regulation of endothelial cell migration depends on urokinase plasminogen activator expression. J. Biol. Chem..

[115] Lamalice L., Le Boeuf F., Huot J. (2007). Endothelial cell migration during angiogenesis. Circ. Res..

[116] Jittreetat T., Shin Y.S., Hwang H.S., Lee B., Kim Y.S., Sannikorn P., Kim C. (2016). Tolfenamic acid inhibits the proliferation, migration, and invasion of nasopharyngeal carcinoma: Involvement of p38-mediated down-regulation of Slug. Yonsei Med. J..

[117] Chiang K., Yang S., Chang K., Feng T., Chang K., Tsui K., Shin Y., Chen C., Chao M., Juang H. (2018). Caffeic acid phenethyl ester induces N-myc downstream regulated gene 1 to inhibit cell proliferation and invasion of human nasopharyngeal cancer cells. Int. J. Mol. Sci..

[118] Liu Y., Chuang Y., Lo Y., Lin C., Hsi Y., Hsieh M., Chen M. (2020). Asiatic acid, extracted from Centella asiatica and induces apoptosis pathway through the phosphorylation p38 mitogen-activated protein kinase in cisplatin-resistant nasopharyngeal carcinoma cells. Biomolecules.

[119] Hsieh M., Wang C., Lin J., Chuang Y., Hsi Y., Lo Y., Lin C., Chen M. (2019). Celastrol, a plant-derived triterpene, induces cisplatin-resistance nasopharyngeal carcinoma cancer cell apoptosis though ERK1/2 and p38 MAPK signaling pathway. Phytomedicine.

[120] Chen Y., Chen P., Lin C., Yang W., Ho Y., Yang S., Chuang C. (2020). Cantharidic acid induces apoptosis in human nasopharyngeal carcinoma cells through p38-mediated upregulation of caspase activation. Environ. Toxicol..

[121] Chuang C., Tang C., Ho H., Hsin C., Weng C., Yang S., Chen P., Lin C. (2019). Licochalcone A induces apoptotic cell death via JNK/p38 activation in human nasopharyngeal carcinoma cells. Environ. Toxicol..

[122] Hsieh M.J., Chien S.Y., Chou Y.E., Chen C.J., Chen J., Chen M.K. (2014). Hispolon from Phellinus linteus possesses mediate caspases activation and induces human nasopharyngeal carcinomas cells apoptosis through ERK1/2, JNK1/2 and p38 MAPK pathway. Phytomedicine.

[123] Vergote I., Heitz F., Buderath P., Powell M.A., Sehouli J., Lee C.M., Hamilton A.L., Fiorica J., Moore K.N., Teneriello M. (2019). A randomized, double-blind, placebo-controlled phase Ib/II study of ralimetinib, a p38 MAPK inhibitor, plus gemcitabine (G) and carboplatin (C) versus GC for women with recurrent platinum-sensitive ovarian cancer. Gynecol. Oncol..

[124] Tawil A., Mellion M., Ronco L., Raines S., Tracewell W., Rahilly A., Rojas A., Hage M., Wagner K., Statland J. (2021). Design of a Phase 2, Randomized, Double-Blind, Placebo-Controlled, 24-Week, Parallel-Group Study of the Efficacy and Safety of Losmapimod in Treating Subjects with Facioscapulohumeral Muscular Dystrophy (FSHD): ReDUX4 (1592). https://clinicaltrials.gov/ct2/show/NCT04003974.

[125] Ding C. (2006). Drug evaluation: VX-702, a MAP kinase inhibitor for rheumatoid arthritis and acute coronary syndrome. Curr. Opin. Investig. Drugs.

[126] MacNee W., Allan R.J., Jones I., De Salvo M.C., Tan L.F. (2013). Efficacy and safety of the oral p38 inhibitor PH-797804 in chronic obstructive pulmonary disease: A randomised clinical trial. Thorax.

[127] Dotan I., Rachmilewitz D., Schreiber S., Eliakim R., Van der Woude C.J., Kornbluth A., Buchman A.L., Bar-Meir S., Bokemeyer B., Goldin E. (2010). A randomised placebo-controlled multicentre trial of intravenous semapimod HCl for moderate to severe Crohn’s disease. Gut.

[128] Messoussi A., Feneyrolles C., Bros A., Deroide A., Daydé-Cazals B., Chevé G., Van Hijfte N., Fauvel B., Bougrin K., Yasri A. (2014). Recent progress in the design, study, and development of c-Jun N-terminal kinase inhibitors as anticancer agents. Chem. Biol..

[129] Wu Q., Wu W., Jacevic V., Franca T.C., Wang X., Kuca K. (2020). Selective inhibitors for JNK signalling: A potential targeted therapy in cancer. J. Enzym. Inhib. Med. Chem..

[130] Patnaik A., Haluska P., Tolcher A.W., Erlichman C., Papadopoulos K.P., Lensing J.L., Beeram M., Molina J.R., Rasco D.W., Arcos R.R. (2016). A first-in-human phase I study of the oral p38 MAPK inhibitor, ralimetinib (LY2228820 dimesylate), in patients with advanced cancer. Clin. Cancer Res..

[131] Cicenas J., Zalyte E., Rimkus A., Dapkus D., Noreika R., Urbonavicius S. (2018). JNK, p38, ERK, and SGK1 inhibitors in cancer. Cancers.

[132] Kim J., Kim T.H., Kang H.S., Ro J., Kim H.S., Yoon S. (2009). SP600125, an inhibitor of Jnk pathway, reduces viability of relatively resistant cancer cells to doxorubicin. Biochem. Biophys. Res. Commun..

[133] Kim J., Chae M., Choi A., Kim H.S., Yoon S. (2014). SP600125 overcomes antimitotic drug-resistance in cancer cells by increasing apoptosis with independence of P-gp inhibition. Eur. J. Pharm..

[134] Lu Y., Chen T., Wang X., Qu J., Chen M. (2010). The JNK inhibitor SP600125 enhances dihydroartemisinin-induced apoptosis by accelerating BAX translocation into mitochondria in human lung adenocarcinoma cells. FEBS Lett..

[135] Lin Y., Zhang B., Liang H., Lu Y., Ai X., Zhang B., Chen X. (2013). JNK inhibitor SP600125 enhances TGF-β-induced apoptosis of RBE human cholangiocarcinoma cells in a Smad-dependent manner. Mol. Med. Rep..

[136] Jemaa M., Vitale I., Kepp O., Berardinelli F., Galluzzi L., Senovilla L., Marino G., Malik S.A., Rello-Varona S., Lissa D. (2012). Selective killing of p53-deficient cancer cells by SP.. EMBO Mol. Med..

[137] Konno T., Ninomiya T., Kohno T., Kikuchi S., Sawada N., Kojima T. (2015). c-Jun N-terminal kinase inhibitor SP600125 enhances barrier function and elongation of human pancreatic cancer cell line HPAC in a Ca-switch model. Histochem. Cell Biol..

[138] Li J., Huang J., Xing B., Ren K., Li M., Wei D., Gu P., Chen G., Gu B., Zhang G. (2012). SP600125, a JNK inhibitor, suppresses growth of JNK-inactive glioblastoma cells through cell-cycle G2/M phase arrest. Die Pharm.—Int. J. Pharm. Sci..

[139] Gao Y., Cheng J., Zeng Q., Xu Z., Decosterd I., Xu X., Ji R. (2009). Selective inhibition of JNK with a peptide inhibitor attenuates pain hypersensitivity and tumor growth in a mouse skin cancer pain model. Exp. Neurol..

[140] Zhang T., Inesta-Vaquera F., Niepel M., Zhang J., Ficarro S.B., Machleidt T., Xie T., Marto J.A., Kim N., Sim T. (2012). Discovery of potent and selective covalent inhibitors of JNK. Chem. Biol..

[141] Ebelt N.D., Kaoud T.S., Edupuganti R., Van Ravenstein S., Dalby K.N., Van Den Berg C.L. (2017). A c-Jun N-terminal kinase inhibitor, JNK-IN-8, sensitizes triple negative breast cancer cells to lapatinib. Oncotarget.

[142] Okada M., Kuramoto K., Takeda H., Watarai H., Sakaki H., Seino S., Seino M., Suzuki S., Kitanaka C. (2016). The novel JNK inhibitor AS602801 inhibits cancer stem cells in vitro and in vivo. Oncotarget.

[143] Posthumadeboer J., Van Egmond P.W., Helder M.N., De Menezes R.X., Cleton-Jansen A.M., Belien J.A., Verheul H.M., Van Royen B.J., Kaspers G.J., Van Beusechem V.W. (2012). Targeting JNK-interacting-protein-1 (JIP1) sensitises osteosarcoma to doxorubicin. Oncotarget.

[144] Vasilevskaya I.A., Selvakumaran M., Hierro L.C., Goldstein S.R., Winkler J.D., O’Dwyer P.J. (2015). Inhibition of JNK sensitizes hypoxic colon cancer cells to DNA-damaging agents. Clin. Cancer Res..

[145] Düzgün Ş.A., Yerlikaya A., Zeren S., Bayhan Z., Okur E., Boyacı İ. (2017). Differential effects of p38 MAP kinase inhibitors SB203580 and SB202190 on growth and migration of human MDA-MB-231 cancer cell line. Cytotechnology.

[146] Yasui H., Hideshima T., Ikeda H., Jin J., Ocio E.M., Kiziltepe T., Okawa Y., Vallet S., Podar K., Ishitsuka K. (2007). BIRB 796 enhances cytotoxicity triggered by bortezomib, heat shock protein (Hsp) 90 inhibitor, and dexamethasone via inhibition of p38 mitogen-activated protein kinase/Hsp27 pathway in multiple myeloma cell lines and inhibits paracrine tumour growth. Br. J. Haematol..

[147] He D., Zhao X., Chen X., Fang Y., Singh S., Talele T.T., Qiu H., Liang Y., Wang X., Zhang G. (2013). BIRB796, the inhibitor of p38 mitogen-activated protein kinase, enhances the efficacy of chemotherapeutic agents in ABCB1 overexpression cells. PLoS ONE.

[148] Jin X., Mo Q., Zhang Y., Gao Y., Wu Y., Li J., Hao X., Ma D., Gao Q., Chen P. (2016). The p38 MAPK inhibitor BIRB796 enhances the antitumor effects of VX680 in cervical cancer. Cancer Biol. Ther..

[149] Hideshima T., Podar K., Chauhan D., Ishitsuka K., Mitsiades C., Tai Y., Hamasaki M., Raje N., Hideshima H., Schreiner G. (2004). p38 MAPK inhibition enhances PS-341 (bortezomib)-induced cytotoxicity against multiple myeloma cells. Oncogene.

[150] Giafis N., Katsoulidis E., Sassano A., Tallman M.S., Higgins L.S., Nebreda A.R., Davis R.J., Platanias L.C. (2006). Role of the p38 mitogen-activated protein kinase pathway in the generation of arsenic trioxide-dependent cellular responses. Cancer Res..

[151] Campbell R.M., Anderson B.D., Brooks N.A., Brooks H.B., Chan E.M., De Dios A., Gilmour R., Graff J.R., Jambrina E., Mader M. (2014). Characterization of LY2228820 dimesylate, a potent and selective inhibitor of p38 MAPK with antitumor activity. Mol. Cancer.

[152] Yeung Y.T., Yin S., Lu B., Fan S., Yang R., Bai R., Zhang C., Bode A.M., Liu K., Dong Z. (2018). Losmapimod overcomes gefitinib resistance in non-small cell lung cancer by preventing tetraploidization. EBioMedicine.

[153] Bachegowda L., Morrone K., Winski S.L., Mantzaris I., Bartenstein M., Ramachandra N., Giricz O., Sukrithan V., Nwankwo G., Shahnaz S. (2016). Pexmetinib: A novel dual inhibitor of TIE2 and p38 MAPK with efficacy in preclinical models of myelodysplastic syndromes and acute myeloid leukemia. Cancer Res..

[154] Wollenberg L.A., Corson D.T., Nugent C.A., Peterson F.L., Ptaszynski A.M., Arrigo A., Mannila C.G., Litwiler K.S., Bell S.J. (2015). An exploratory, randomized, parallel-group, open-label, relative bioavailability study with an additional two-period crossover food-effect study exploring the pharmacokinetics of two novel formulations of pexmetinib (ARRY-614). Clin. Pharm..

[155] Chen J.C., Hsieh M.J., Chen C.J., Lin J.T., Lo Y.S., Chuang Y.C., Chien S.Y., Chen M.K. (2016). Polyphyllin G induce apoptosis and autophagy in human nasopharyngeal cancer cells by modulation of AKT and mitogen-activated protein kinase pathways in vitro and in vivo. Oncotarget.

[156] Roulston A., Reinhard C., Amiri P., Williams L.T. (1998). Early activation of c-Jun N-terminal kinase and p38 kinase regulate cell survival in response to tumor necrosis factor α. J. Biol. Chem..

